# Outcomes after Hallux Rigidus surgery: Are we measuring what is important to patients? A qualitative analysis of patient-identified outcomes

**DOI:** 10.1371/journal.pone.0332385

**Published:** 2025-09-15

**Authors:** Alison W. Henderson, Sienna L. Williams, Jay Crary, Bruce Sangeorzan, William Ledoux, Pamela Fairchild, Daniel C. Norvell

**Affiliations:** 1 VA Puget Sound Health Care System, Seattle, Washington, United States of America; 2 Rebound Orthopedics and Neurosurgery, Vancouver, Washington, United States of America; University of Illinois Urbana-Champaign, UNITED STATES OF AMERICA

## Abstract

**Background:**

Hallux rigidus (HR) is the most common form of arthritis in the foot. The joint pain and loss of motion from HR experienced during walking may lead to a significant reduction in activity and quality of life, and advanced cases may require surgery. HR surgical outcomes are often evaluated quantitatively with more generic measures not designed specifically to assess HR outcomes. The goal of these study was to determine outcomes that are important to patients with HR, possibly those that are not addressed by more general foot and ankle measures.

**Methods:**

Semi-structured interviews with HR surgical patients 4–9 years after surgery were conducted. Interviews were analyzed using a team-based, iterative inductive-deductive approach to identify outcomes important to patients.

**Results:**

Ten patients were interviewed; five who received motion-sparing surgery and five with fusion surgery. From interviews, seven themes were identified: pain, first MTPJ motion, walking ability, physical activity, footwear, forefoot appearance, and pain in other areas of the body.

**Conclusions:**

Post-HR surgery patients indicated outcomes of importance that are not addressed by more general instruments. Specifically, forefoot appearance and pain in other areas of the body are not addressed by commonly used instruments. In addition, patient experiences of pain are more granular and HR-specific than the generic pain items used by other instruments. Patients facing HR surgery may benefit from outcome measures that are more specific to the HR surgical experience and include outcomes that patients share are important to them that are not included in commonly used outcome instruments.

## Introduction

Hallux rigidus (HR), a degenerative disease of the first metatarsophalangeal joint (MTPJ), is the most common form of arthritis in the foot, affecting 1 in 40 people over the age of 50 [1,2]. The population prevalence of symptomatic osteoarthritis of the first MTPJ has been reported as high as 7.8% [3]. The first MTPJ plays a critical functional role in walking as it carries an estimated 119% of an individual’s body weight with each step [4]; therefore, the joint pain and loss of motion experienced during walking may lead to reduction in activity and quality of life [5,6]. HR is typically treated by shoe and activity modifications and NSAIDs. When these treatments are ineffective, surgery may be an option.

HR surgical outcomes are often evaluated quantitatively, using a variety of measures. To date, there are few HR-specific patient-reported outcome measures; therefore, patients are often evaluated with more generic measures or orthopedic measures not designed specifically to assess HR outcomes. One of the most commonly used measures is the American Orthopedic Foot and Ankle Society-Hallux Metatarsophalangeal Interphalangeal (AOFAS-HMI) score [7,8]. The AOFAS-HMI score is a frequently-used measure that evaluates pain, function, and alignment [9]. Another frequently used measure is the Foot and Ankle Ability Measure (FAAM) [10], which addresses a more broad range of domains related to both foot and ankle disability; however, it does not evaluate specific first MTPJ functional disability. Other than footwear challenges, it is unclear if there are other issues that patients with HR face that are not addressed by more general foot and ankle measures.

As the field of medicine moves toward a more patient-centric focus, there is a push to emphasize issues that are most relevant and important to patients themselves. Many outcome measures, even patient-reported outcome measures (PROMs) fail to actively incorporate patients in the development process [11–14]. Only direct patient engagement, including qualitative methods, will uncover whether the domains measured in existing foot/ankle outcome measures adequately assess what is important to HR patients. The purpose of this study was to determine what outcomes are important to patients after HR surgery and to determine if existing PROMs are adequate in measuring these outcomes, or if there are additional domains identified as important. The ultimate goal is to use the themes identified in this study to develop a future HR PROM that more specifically measures patient outcome priorities after surgery.

## Materials and methods

### Participants

A subset of 32 individuals who had undergone HR surgery and had previously participated in a larger pilot study evaluating HR surgical outcomes were approached to participate in qualitative interviews. The pilot study results were used for an NIH grant proposal and are not published elsewhere. Of the 117 enrolled in the pilot study, 32 participants agreed to be contacted to participate in future studies, and these individuals were recruited for qualitative interviews. Those who had not responded to the initial letter after two weeks were contacted by phone. During follow-up calls, a researcher described the study and answered questions prior to enrollment. Written and oral consent were obtained for different aims of study. Documentation of consent was waived. All subjects provided verbal informed consent, and this study was approved by a commercial institutional review board (Advarra; Pro00048033).

### Interviews

Open-ended interviews (see Appendix) were conducted to attempt to answer the following questions: (1) what outcomes were important before pre- and post-surgery, as well as what might be important in the future; and (2) what post-surgical experiences shaped their current outcome and quality of life. Interviews were conducted via telephone, digitally recorded, transcribed, and stored as audio files on a secure drive. Analyses and summary manuscripts were performed by a trained qualitative analyst.

### Data Analysis

Interviews were conducted until researchers determined data saturation was reached, when new information did not contribute significantly to our research question or when data presented were redundant to those already collected [15,16]. Content analysis of interview transcripts was performed using an iterative inductive-deductive approach [17]. Codes were attached to transcript passages representing themes or sub-themes. Atlas.ti software (v.23, Berlin) was used to organize interview data and present it by surgery type and time window. Codebook consensus was reached by two analysts individually coding the same four transcripts and discussing overlap and gaps between their datasets. Initial code categories were developed from factors related to HR outcomes identified in the literature [8]. Inductive codes were created to identify key findings and were added throughout coding after discussion between analysts. Analysis results were synthesized and reviewed by an expert panel of surgeons, an epidemiologist with experience in outcome measure development, an implementation scientist, two research coordinators with experience administering outcome measures to patients with HR, and a mechanical engineer with research experience in HR biomechanics. Given that quantitative outcome measures are commonly used to evaluate changes in function and differences between interventions, we have organized the qualitative results to note changes between pre- and post-hallux surgery, whenever they were observed for a given theme. Similarly, we have noted differences between the two surgery groups if qualitative analyses noted important differences in patient experiences.

## Results

### Participants

Eleven individuals consented to participate and completed the interview. One patient’s interview data was not recorded, due to a technical error, therefore data on 10 patients were analyzed. At completion of these interviews, it was determined that data saturation was reached, as there were no new data being presented. As such, no additional participants were enrolled for participation. These 10 patients were mostly male (70%) and the average time since HR surgery was greater than 7 years (range, 4–9 years). The enrolled sample was evenly distributed between those who received motion sparing and those who received fusion surgeries (Table 1). Among the motion sparing patients, there were three cheilectomies and two arthroplasties. We also describe those enrolled in the pilot study who did not consent to be contacted for subsequent participation to ensure that the sample was representative of the larger population (n = 85; Table 1). There were few significant differences between those who enrolled in the qualitative study and those who were enrolled in the pilot study. Most notably, a greater proportion of males enrolled in the qualitative study than in the pilot study. Also, participants who enrolled in the qualitative study had a significantly more proximal surgery date relative to the pilot study population (Table 1).

**Table 1:** Participant characteristics comparing participants enrolled and the larger pilot study population.

	Motion Sparing (N = 5) N (%) or M (SD)	Fusion (N = 5) N (%) or M (SD)	All Enrolled (N = 10) N (%) or M (SD)	Not enrolled (N = 85) N (%) or M (SD)	P-value^***^
Age	56.0 (6.8)	6.8 (9.8)	60.8 (9.2)	58.1 (10.5)	P = 0.42
Fusion surgery	0 (0%)	5 (100%)	5 (50%)	48 (56%)	P = 0.73
Male	4 (80%)	3 (60%)	7 (70%)	27 (32%)	P = 0.04
White	4 (80%)	5 (100%)	9 (90%)	82 (96%)	P = 0.21
Married	4 (80%)	3 (60%)	7 (70%)	63 (74%)	P = 0.78
At least some college	3 (60%)	4 (80%)	7 (70%)	77 (91%)	P = 0.28
Currently employed	1 (20%)	3 (60%)	4 (40%)	48 (56%)	P = 0.41
Years since surgery	7.8 (1.5)	6.48 (1.8)	7.14 (1.7)	8.36 (1.1)	P = 0.002
Functional Comorbidity Index total score	2.4 (1.7)	2.8 (1.9)	2.60 (1.7)	2.45 (1.6)	P = 0.98
FAAM ADL score^*^	53.51 (21.9)	63.81 (23.7)	58.66 (22.2)	66.9 (18.1)	P = 0.26
FAAM Sport score^*^	38.32 (22.96)	29.60 (27.74)	34.35 (24.3)	39.3 (22.7)	P = 0.51
Current foot pain intensity^**^	3.8 (2.6)	4.8 (2.5)	4.3 (2.5)	4.1 (2.6)	P = 0.94
Most intense foot pain in last 6 months	9.0 (0.7)	7.6 (2.3)	8.3 (1.8)	8.0 (2.1)	P = 0.95
Average foot pain intensity in last 6 months	5.6 (1.8)	5.8 (2.2)	5.7 (1.9)	5.5 (2.2)	P = 0.89
Average overall pain intensity in last 6 months	6.4 (2.7)	5.0 (2.2)	5.70 (2.5)	4.8 (3.0)	P = 0.58

* The higher the score the greater the function (0–100 point scale).

** All pain intensity measured by a 0–10 point visual analogue scale.

*** Significance testing between enrolled and not enrolled was conducted using Chi-square for categorical variables or t-test for continuous variables.

### Themes

Seven themes were identified after data saturation was met. The influence of pain was noted as the primary driver for seeking out HR surgery, specifically its effect on motion of the effected toe, mobility, activities of daily living and footwear. Several patients also discussed changes in the appearance of their forefoot after HR surgery, as well as additional pain in other areas of their body. The following sections summarize the themes identified, and quotes supporting themes are delineated to note those who underwent a motion sparing (ms) and fusion (f) surgery (Table 2). In addition, we reference the presence of these themes in our work and in the commonly used instruments noted above (Table 3).

**Table 2:** Patient quotes to support identified themes.

Theme	Subtheme	Supporting patient quotes
**Pain**	Pain as a motivator to undergo HR surgery	*“Mostly just a lot of pain, like constant.”* (ms2004) *“It was very painful. Even if I wasn’t doing anything it was still painful.”* (f2054) *“No pain. It’s I guess what everybody wants, isn’t? I can’t imagine what else I could think of besides not suffering.”* (ms2036) *“Yeah, just to walk without pain.”* (f2014)
	Reduction in pain post HR surgery	“*I mean the pain was really bad before I had it, and now I can easily live with the small things I get occasionally.”* (ms2036) *“I’ve got no complaints. Except it still kind of hurts, but that’s not really a complaint. I can live with it.”* (ms2002) *“Oh, well, after the pain of the surgery went away, all of the other pain in my toe went away. So that was great. That’s what I was hoping for, and that’s what happened.* (f2054)
**First MTPJ motion**	Prior to HR surgery, pain reduced motion in toe	*“I could walk, but I couldn’t bend my toe the way, you know when you walk, your toe bends the way it’s supposed to. Mine didn’t do that, and it hurt. The bones were, I don’t know, growing together. They were just like stuck…..”* (ms2002) *“You know that toe, it bends a certain amount. Before I had the replacement put in, I think you avoid trying to bend it because you’re avoiding causing any more pain. This one, I can push off of it a certain amount, without any pain.”* (ms2004) “*Well, I just remember my toe was giving me a lot of pain, and I was really walking on the outside of my shoes and wearing them out. Because I was rolling off of the toe, so it didn’t try to bend or anything because it was so painful.”* (ms2004) *“I didn’t know from one day to the next how my toe would respond… Sometimes just the thought of having to move that toe was concerning, because there could be a lot of pain, or there might not be.”* (ms2024) *“Because I wanted to be as active as I could. And I wanted that joint to be able to move. So, I would do whatever it would take to do one where the joint could continue to move.”* (ms2024)
	Increased motion in toe among MS patients post HR surgery	*“… I take my shoe off and wiggle my toes”* (ms2002) *“… gradually my toe became more and more pliable or had movement.”* (ms2024) *“Yeah, it was just… not as movable as I thought it was going to be.”* (ms2004)
	Decreased motion in toe among fusion patients	*“You don’t realize how much you use your toes; you know? You can’t do yoga. Yoga means you’re getting up and down, and up and down off the floor. You don’t realize how much you use your toes when you get up and down off the floor. You know? You use your toes a lot.* (f2023) *“But I’m not able to move my toe at all, it sticks. It’s not moving… it just stays in one position.” (f2014)* *“… even though I lost mobility of my toe, but I don’t care. As long as it doesn’t hurt.”* (f2014) *“… I really didn’t know what to expect as far as how limiting it would be. But I was willing to accept I think just about any limitation to get rid of that constant pain.”* (f2054)
**Walking ability**	Prior to HR surgery, pain interfered with walking ability	*“Well, it was very painful to walk or put any pressure on it.”* (ms2037) *“It was like walking in tight shoes, and stepping on like a bad sore or something like that, you know? It was painful.”* (f2014) *“I mean, I needed to get it done so I could walk normal. I used to work at Target, so I used to walk everywhere, so I needed to move.”* (f2014) *“…I used to love walking.”* (f2014)
	Decreased pain interference after HR surgery	*“Well, I had hoped to be able to walk without pain, and that has happened, so I’m quite happy with it now…I’m able to walk and carry out activities of daily living that were hampered before by pain. It was difficult to move around, and now I can walk pretty much pain free. So that’s a major thing in my life.”* (ms2037) *“I was able to walk, and pretty much to walk fast. I used to walk around a park, so I could do exercise. It was like, maybe like 5 miles around. I used to do that like, one day yes, and one day no. Every other day.”* (f2014) *“Walking downstairs, for me, I need to be a lot more cautious doing that than I used to. Walking upstairs, that’s not bad, but going downstairs, I pretty much, hopefully there’s a handrail…”* (f2054)
**Physical activity**	Desire to increase physical activity as a motivator to undergo HR surgery	*“But as far as my own actions, I love to run and play tennis, and it was affecting my ability to do both of those.”* (ms2024) *“I’m pretty active, and I was backpacking, running and cycling. Although cycling didn’t bother me, it was the impact sports that were painful. And that went on for several years, actually….”* (f2025)
	Patient satisfaction in ability to return to higher levels of physical activity after HR surgery	*“Well, I can work out at the gym 5 days a week…I mean before I had the surgery, I was trying to figure out a way to even walk on it without it hurting.”* (ms2004) *“Because I wanted to be as active as I could. And I wanted that joint to be able to move. So, I would do whatever it would take to do one where the joint could continue to move.”* (ms2024) *“I think I got everything I wanted out of it. If I can’t run or play tennis, well I go out and play tennis sometimes, but if I can’t run, it’s based on the pain on the outside of my leg, it has nothing to do with my foot.”* (ms2024) *“Oh, so much better. Yeah, I’m able to, now I devote most of my time to athletic activity and working out, and that includes things I do with my legs. I wouldn’t be able to do some of those had I not had toe surgery. And then just in normal day to day life, trying to figure out what shoes are going to be ok for me to walk in, and how long I’ll be able to walk without my toe really bothering me. I don’t have any of that anymore.”* (ms2024) *“Well, I realized I could go hiking in the Dolomites, and go on a backpacking trip in the Artic, which I’ve done. I wouldn’t have been able to do those without the surgery.”* (f2025)
	Concerns around engaging in physical activity after HR surgery	*“…would I do it all over again? Yeah. But it has really limited my ability to kind of just be normal. Prior to that, I never had to favor and think about if I step wrong, I’m going to break by toe, basically. And now I constantly have to think like that…It’s because it’s a lifestyle change, you know? It’s limiting now. If it’s a person that was a little bit active, and they’re suffering with that pain, say they like to go on hikes and stuff like that, well they’ve really got to reconsider that lifestyle following that surgery. I’m not saying that a person can’t do it, but not at the level they once did, no way.”* (f2054)
**Footwear**	Prior to HR surgery, pain limited footwear options	*“Painful and limited…Shoes. I couldn’t wear, I was very limited with what I could put on my feet.”* (f2030) “*Oh, it was very painful all of the time. I couldn’t wear shoes.”* (ms2036)
	Some continued to experience footwear limitations after HR surgery	*“…I have a problem if I wear shoes that are too tight, and you learn once. But if I put on a pair of shoes that are too tight and I wear them for any length of time, then my toe will be in pain, the toe that had the surgery, where the other toe does not.”* (ms2024) *“Yeah, I cannot wear heels or hard shoes…Sometimes, because I still have some shoes, I haven’t thrown them away or anything, I just like them…But I cannot wear them because they’re too hard.”* (f2014) *“So, I was really miserable with my recovery because I couldn’t find shoes that would work for me, even after my foot healed. So, I was not happy. I wore my boot much longer than I needed to because I couldn’t find shoes. I was looking all over. So, I was not happy. I was happy not to be in pain, but I couldn’t find any shoes that would work.”* (f2023) “*Yeah, heels, I can’t really wear heels. But at my age…I don’t need to be in high heels either, my kids would probably kill me, they’d say, you’re going to fall and break your neck.”* (ms2004)
**Forefoot appearance**	One patient indicated forefoot appearance as a motivator to undergo HR surgery	*“I had big lumps on both feet. It was painful, it was kind of weird. There was some discomfort to it. And it was ugly, that type of thing. It hurt sometimes; it was hard to wear shoes.”* (f2056)
	Improvement in forefoot appearance after arthrodesis procedure	*“But all in all, it was the best option, because right now I don’t have big lumps on my toes, and I don’t have the discomfort I had before.”* (f2056) *“The discomfort, pain, and getting rid of the ugly feet. And it did everything…I am completely happy with both surgeries, and I’m glad I did it…It was well worth it.”* (f2056)
	Motion sparing procedure worsened forefoot appearance	*“I went back…and…had a follow up with him. It seems to me that my big toe was not put back in the exact right position so now it’s overlapped by my second toe…I had an appointment, but, I must say, he was rather brisk with me. He said, well, I think your toe was in that position before we started. I said, oh, well, alright. So, I let it go.”* (ms2036)
**Pain in other areas of the body**	Patients experience pain in other areas of their body, not attributed to HR surgery	“My toe. It doesn’t stop me from walking, no. It’s my knees. I have a tear. They told me I have a slight tear in my right knee, but they won’t fix it because I weigh too much. I’m ***AGE*** years old, so I guess I’m going to have to just eat this one.” (ms2002) *“No, but I think it’s expanding my arthritis, I mean it came from one place and then moved to my knees, from my knees, it moved to my hip. And my other hip started bothering me now, again. And I have a problem with my bone density in my mouth, with my gums. And that’s because of arthritis.”* (f2014)
	Patients experience pain in other areas of their body, and attribute to HR surgery	*“… what I didn’t find helpful was, before surgery, and after, there was never any mention of, you’ve been favoring that foot for 20 something years, and what I believe, because of that, I could have knee issues, I could have hip issues. ….”* (f2025) *“I don’t notice it as much, but my gait is definitely a little different... where the weight is on my foot, it offsets the rest of your body, how you’re putting weight on your foot… it’s just your body is always compensating, because we’re messing with nature a little bit.* (f2030)

HR = hallux rigidus; ms = motion sparing; f = fusion

**Table 3:** Factors identified as important in this study and instruments commonly used to evaluate hallux rigidus outcomes.

Outcome	This study	AOFAS-HMI score	FAAM
Pain	X	X	
First MTPJ motion	X	X	
Walking ability	X		X
Physical activity	X		X
Footwear	X	X	
Forefoot appearance	X		
Pain in other areas of the body	X		
Activities of daily living		X	X
Alignment		X	

AOFAS-HMI score: American Orthopedic Foot and Ankle Society-Hallux Metatarsophalangeal Interphalangeal score; FAAM: Foot and Ankle Ability Measure.

### Theme 1: Pain

Nearly all patients reported experiencing significant pain prior to HR surgery, experiences that did not appear to vary among the two surgical groups. Both groups reported nearly constant pain prior to surgery, and cited alleviation as a primary motivator for seeking surgical intervention. While some indicated that they still experienced pain in their toes following surgery, all patients noted that they experienced significantly less pain than they did prior to surgery, an experience that did not appear to differ between the motion sparing and arthrodesis patients.

### Theme 2: First MTPJ motion

Prior to surgery, several patients (all patients who received motion sparing surgery) recalled HR pain limited motion in their toes. Post HR surgery, many commented on the changes in toe movement. Several, but not all, motion sparing patients recalled increased toe movement. In contrast, many fusion patients noted that toe movement decreased, one patient to great frustration. Several others noted this loss of mobility was acceptable given the reduction in pain.

### Theme 3: Walking ability

Prior to their HR surgery, many patients found that their HR pain significantly interfered with their walking ability. Post surgery, patients reported little to no pain, although one patient noted that, post-fusion surgery, walking downstairs posed a specific challenge.

### Theme 4: Physical activity

More than just simply walking without pain, some patients indicated a desire to return to their once active lifestyles by undergoing HR surgery. Following surgery, patients commented on their ability to return to high levels of physical activity. Some patients did note that physical activity might be limited post-surgery due to concern about injuring bones at the surgical site, especially those undergoing fusion.

### Theme 5: Footwear

Regardless of surgical intervention, many patients expressed that prior to HR surgery, they were limited in the types of shoes they could wear, primarily due to pain wearing certain shoes. Some patients indicated that they did feel limited in the types of shoes they could wear following HR surgery, an experience reported by patients who underwent motion sparing as well as arthrodesis surgery, and some expressed disappointment with footwear restrictions or limitations, whereas the inability to wear certain types of shoes, like dress, or heels was no longer a primary cause for concern for others.

### Theme 6: Forefoot appearance

Several patients volunteered more information about the look and feel of their toes both pre- and post-surgery. For one fusion patient, improving the physical appearance of their feet and toes was a motivator for surgery, and that surgery did improve forefoot appearance. Another motion sparing patient experienced disappointment when speaking with a doctor after their surgery about a change in appearance to their toes, which they felt was due, at least in part, to their HR surgery.

### Theme 7: Pain in other locations on the body

A significant number of patients shared that they continued to experience pain that interfered with their walking ability after HR surgery. However, they noted that this pain was no longer concentrated in their HR affected toes, but rather, to other areas of their body. Some individuals understand these limitations as unrelated to their HR, but rather the progression of arthritis in other areas of the body as they age. Other patients attributed the pain in other areas of their body to gait changes, both due to their HR, as well as surgery to correct HR.

## Discussion

In interviews with 10 patients post-HR surgery, seven themes were identified: pain as a primary driver for surgery, first MTPJ motion, walking ability, engagement in physical activity, footwear, appearance of the toe, and pain in other locations of the body.

While pain was a universal motivation for surgery, other identified themes played varying roles in their assessment of the impact of HR on their lives preoperatively and the success of the surgery postoperatively. For example, shoe wear limitation may be substantial in two different patients, however, that limitation may be very important to one and inconsequential to another. Understanding this patient-specific variability will allow for improved decision making.

The development and use of PROMs has increased in recent years, particularly with the development of the PROMIS tools [18]. However, while there are several knee-specific osteoarthritis measures, but no general measures, and no HR-specific measures [19]. In absence of an established HR-specific measure, several existing measures are reported in the literature evaluating HR outcomes. Although the AOFAS-HMI score is frequently used to assess HR surgical outcomes, it has a noted limitation. It has been found to have poor construct validity relative to the SF-36 (often used as a benchmark for orthopaedic measures) [20]. In light of this, the AOFAS published a position statement in 2011 indicating that they recommend the AOFAS scales no longer be used [21]. Despite this publication, the AOFAS scales continue to be some of the most frequently used in foot and ankle research, and are regularly used in published research and recommendations [22]. While the AOFAS-HMI score evaluates pain, physical activity and footwear, it does not include the domains of mobility, motion, or appearance of the toe. Further there are only three questions on footwear, and they are limited to requirements (e.g., whether or not a shoe insert or brace is necessary), and do not address patient satisfaction with these requirements. Our research group is currently conducting a multisite National Institute of Health study comparing fusion to various motion sparing techniques and had an expert panel create several footwear question due to this limitation. In addition, completion of the measure (evaluation of alignment) requires clinician input which increases burden, so it cannot be completed exclusively by patients. Another frequently used measure is the FAAM [10]. The FAAM includes a 21-item activities of daily living (ADL) subscale and an 8-item Sports subscale, which can be combined to describe self-reported function. The FAAM was developed by studying patients who were undergoing physical therapy for varied leg, ankle, and foot conditions, including forefoot disorders. While patients in this study described some experiences that map onto FAAM domains (mobility and physical activity), additional domains were mentioned by patients that the FAAM does not measure: pain, toe motion, forefoot appearance and footwear. Further, the FAAM does not evaluate specific disability caused by the first MTP joint, and, as such, may be too generic for evaluating HR outcomes. Thus, there appears to be no currently published measure that addresses all themes identified by this patient population.

In a review of 189 studies describing the development of 193 PROMs, patient involvement in determining which outcomes to evaluate was 11% [14]. In fact, in over 25% of the studies evaluated, there was no patient involvement in the measure development at all. In addition, there is increasing data supporting that orthopaedic operative recovery and rehabilitation are influenced by patient factors that may not be sufficiently captured in PROMs [12], and that preoperative mental health may influence patient patient-reported outcomes post-orthopaedic and post-HR surgery [11,13], demonstrating the need to ensure that outcome measures fully encapsulate a patient’s experience.

A qualitative study interviewing patients with HR and/or hallux valgus (HV) who were recommended for surgery (7 of the 16 patients were diagnosed with HR) identified three major themes: “the impact of pain,” “the surgical decision-making process,” and “body image, the self and identity” [23]. Patients described forefoot pain as debilitating, which impacted their mobility, interfered with activities of daily living, and impacted patients’ mood. Patients also voiced hope that surgery would minimize pain, and that they anticipated returning to “normal life” after surgery. Finally, female patients reported gaining weight because of limited activity due to forefoot pain, as well as limited footwear. It should be noted that this study did not separate those with HR from those with HV when describing themes, so it is not possible to identify if the themes identified were representative of only HR patients. This same group published a study of 15 patients post-HR or HV surgery (6 patients with HR), conducting qualitative interviews at 6-, 12- and 18-months post-surgery. Across interviews, five themes were identified: physical limitations, the psychosocial impact of recovery, regaining a sense of normality, expectations regarding physical recovery and a changed body-image [24]. Some patients reported that while the pain that drove their decision to undergo surgery had abated, changes to gait or posture post-surgery led to pain in new locations on their body, consistent with our findings. Shortly after surgery, many patients described depressed mood because of pain and disrupted sleep, but that after healing, noted improvements in mood. For those without complications that prevented successful healing, patients reported appreciation at a return to “normal” life; those who had complications voiced frustration that they had yet to experience a resumption of more usual activities and functioning. Women reported improved body image after being able to resume physical activity and had fewer footwear restrictions. As in their earlier study, the two groups (HR and HV) were not separated in reporting of results, nor were changes over time noted, making these results difficult to anchor and generalize.

Recently, a manuscript addressing the development of an HR-specific PROM was published [25]. This measure includes 10 items, identifying postoperative pain, wearing non-heeled shoes, wearing heels, standing for more than 30 minutes, walking, running, engaging in exercise and in sports, climbing ladders and driving as outcomes of interest. It should be noted that the items were derived by the research team, and while patients were solicited for input about the factors, it was not a patient-driven exploration process. It also should be noted that several of the outcomes of importance we identified in our study (motion and appearance of the toe) are not included in this proposed measure. Finally, the patient sample upon which this measure was developed included only patients who had undergone fusion; additional research will need to be completed to determine if it captures and measures outcomes important to patients receiving motion sparing surgery.

### Study limitations

Given the span of time between HR surgery and the interview (on average, at least 7 years), there is the possibility of recall bias. While this is unavoidable with the passage of time, it is notable that many patients were able to describe the changes in their experience pre- and post-HR surgery; the overall experiences of HR and its surgical consequences do appear salient to patients. It should also be noted that the implications of recall bias are difficult to estimate both in magnitude and in qualitative impact. Although themes were relatively consistent across the study population, the small study size and parameters of the interview guide limit the ability to identify important clinical concerns that may be present in smaller percentages of patients or in patients who have not yet undergone HR surgery. Our study population was 70% male, despite females being diagnosed with HR at twice the rate of males [26]. There may be gender-specific concerns not adequately captured in our analysis. The study participants were also 90% white and therefore lacked ethnic diversity. Though our sample appears representative of the larger cohort form which they were recruited, it should be noted that small sample size of the study participants may obscure some differences that might appear in a larger cohort. Surgical procedures for HR have evolved in the past decade; it may be that a more contemporary population will have somewhat different experiences with their HR surgeries. Future studies may evaluate differences in patient experiences over time. Finally, this sample included HR patients who received surgical treatment from a university medical center in the Pacific Northwest. While there are no specific patient factors that are notably different from the general HR population, it is possible that some facets of patient experiences may vary across regions, as well as among patients who do not seek out surgical intervention to treat their HR symptoms.

## Conclusions

Despite pain being the primary driver for HR surgery, several measures reported in the literature to evaluate HR surgical outcomes and success do not include pain questions. Existing studies often add generic pain questions alongside another non-HR specific functional outcome measure. Pain questions developed for a future HR PROM measure should be specific to the first MTPJ and granular enough to pick up on the pain-specific issues that these patients face. Since HR surgery may alter the gait mechanics of the patient, pain may develop in other joint areas; therefore, additional pain measures assessing other areas should be included. Further, a future measure should include questions specific to walking mobility, including specific mobility tasks that patients have identified as challenging (e.g., walking down steps). After pain, motion was the next most dominant concern of patients. Patient perception of their motion relative to healthy feet and their perception of the resultant impact of that motion should be assessed. Additionally, foot appearance, footwear limitations and impact of a patient’s HR surgery on their mental health should be evaluated. Although pain alleviation was a universal primary goal, the importance of the other themes to the patient, even when abnormal, needs to be understood. The relative importance of the identified themes needs to be understood for each patient facing HR surgery; a similar future study may explore domains of importance in non-operative HR patients. Finally, we recommend the measure be a stand-alone PROM that can be completed by the patient without the requirement of provider evaluations such as range of motion or imaging to reduce the clinical burden.

## Appendix 1. Interview guide questions

### Decision-making prior to surgery

1. Before surgery, what was it like living with big toe arthritis?
  What led you to seek care for your toe? (pain/ difficulty performing ADL/ difficulty at work/ other)
2. Please tell me about the time when you realized you needed toe surgery.
  Where were you?
  Who was there?
  How was it brought up?
3. Did you reach an opinion about the type of surgery (fusion or a procedure that would allow your toe to continue to move, I.e., “motion sparing”) you wanted before meeting with a surgeon?
  What was your main source of information?
4. Was there a discussion about surgical options with your surgeon (fusion versus motion sparing)? If so, tell me about it.
  Did you discuss the pros and cons of the different surgeries (fusion versus motion sparing)?
5. Can you please describe any additional information that would have been helpful when you were making the decision about which type of toe surgery to have?
6. How did you feel about your level of involvement in the decision around the type of surgery to have (fusion vs motion sparing)?
  Would you want to have been more or less involved? (why or why not?)

### Care and experiences after surgery

1. Did you receive any information about what your recovery would be like?
  [If yes] What were you told? How was that information provided?
2. What parts of your rehabilitation experience were positive/helpful?
3. What parts of your rehabilitation experience do you wish were different?

### Priorities/concerns around surgery

1. Describe what you hoped undergoing (fusion or motion sparing) surgery would achieve.
  goals/ADL/footwear
2. How have your hopes/goals changed, now that you are [state the number] years past surgery?
3. Describe any concerns you had about undergoing (fusion or motion sparing) surgery.
4. What concerns do you have about the future of your toe?
5. How has your toe surgery affected your quality of life?
